# Polydatin impairs mitochondria fitness and ameliorates podocyte injury by suppressing Drp1 expression

**DOI:** 10.1002/jcp.25943

**Published:** 2017-04-27

**Authors:** Zheng Ni, Liang Tao, Xu Xiaohui, Zhao Zelin, Liu Jiangang, Song Zhao, Huo Weikang, Xu Hongchao, Wang Qiujing, Li Xin

**Affiliations:** ^1^ Shenzhen Hospital of Southern Medical University Shenzhen Guangdong PR China; ^2^ College of Stomatology of Guangxi Medical University Nanning Guangxi PR China; ^3^ Pharmaceutical College Guangxi Medical University Nanning Guangxi PR China; ^4^ Department of Neurosurgery, Zhujiang Hospital Southern Medical University Guangdong Province China

**Keywords:** diabetic nephropathy, dynamin‐related protein 1, polydatin

## Abstract

Polydatin (PD), a resveratrol glycoside, has been shown to protect renal function in diabetic nephropathy (DN), but the underlying molecular mechanism remains unclear. This study demonstrates that PD stabilize the mitochondrial morphology and attenuate mitochondrial malfunction in both KKAy mice and in hyperglycemia (HG)‐induced MPC5 cells. We use Western blot analysis to demonstrate that PD reversed podocyte apoptosis induced by HG via suppressing dynamin‐related protein 1 (Drp1). This effect may depend on the ability of PD to inhibit the generation of cellular reactive oxygen species (ROS). In conclusion, we demonstrate that PD may be therapeutically useful in DN, and that, podocyte apoptosis induced by HG can be reversed by PD through suppressing Drp1 expression.

## 1 INTRODUCTION

Diabetic nephropathy (DN) is a microvascular complication of diabetes that leads to end‐stage renal disease (Chen et al., 2015 ). Podocyte damage plays a crucial role in the development of DN. Podocytes are highly specialized glomerular epithelial cells with high energetic demands (Blattner et al., 2013 ). An increasing number of studies has demonstrated that mitochondrial fission is closely related to podocyte injury (Chen, Meng, & Zhang, 2013 ). Mitochondrial fission can induce the overproduction of oxygen species (ROS), which causes damage to mitochondrial DNA (mtDNA) and the oxidation respiratory chain (Quinzii et al., 2010 ). Additionally, our previous work has shown that mitochondrial fission is an important factor contributing to podocyte injury in KKAy mice (unpublished data). Drp1 is known to play an essential role in mitochondrial fusion and in promoting high glucose (HG)‐induced podocyte apoptosis (Katusic & Austin, 2014 ). Many reports indicate that under hyperglycemic conditions, Drp1 activity, mitochondrial fission, cytochrome C release, and ROS production are enhanced, thereby promoting cell apoptosis (Minai & Yeheskely‐Hayon, 2013 ).

Polydatin (PD), a resveratrol glycoside extracted from the roots of Polygonum cuspidatum, is widely applied in traditional Chinese remedies (Jiang et al., 2017 ). PD exhibits more potent antioxidant effects than resveratrol due to its specialized biological properties resulting from conformational differences relative to resveratrol (Lanzilli et al., 2012 ). Previous studies have demonstrated that PD can improve heart function, protect against Alzheimer's disease, and ameliorate renal injury via mitochondrial protection (Jin, Xu, Wang, Xu, & Zhang, 2009 ). Furthermore, many studies have suggested that PD suppresses fibronectin accumulation in hyperglycemic glomerular mesangial cells and ameliorates renal function in diabetic rats, thereby inhibiting renal fibrosis in DN (Gao et al., 2015 ). Although, PD exhibits demonstrable mitochondrial protective effects, its regulation of HG‐induced activation of Drp1 in DN remains to be further elucidated.

KKAy mice with spontaneous type 2 diabetes are a widely used animal model in DN research. These mice feeding on a high‐fat diet would show clinical manifestations of hyperglycemia, impaired glucose tolerance, hyperinsulinemia, moderate obesity, hyperlipidemia, and proteinuria. Kidney damage in these mice is very similar to that which occurs during human DN.

In the current study, we used KKAy mice and a conditionally immortalized mouse podocyte cell line (MPC5) to study the protective effect of PD. We demonstrate for the first time that PD strongly protects podocytes in DN by inhibiting Drp1 activation and Drp1‐mediated mitochondria fission.

## 2 MATERIALS AND METHODS

### 2.1 Reagents and antibodies

Anti‐nephrin, anti‐podocin, anti‐cytochrome c, anti‐Caspase3, Anti‐p‐Drp1, anti‐Drp1, and anti‐β‐actin antibodies were obtained from Sigma (Sigma–Aldrich, St. Louis, MO). Anti‐opa1 were purchased from Santa Cruz (Santa Cruz Biotechnology, Santa Cruz, CA). MitoTracker Red ROS was purchased from Sigma (Sigma–Aldrich). PD used for the cell experiments was obtained from Solaibao Company (Beijing, China); PD used for animal treatments was purchased from Mansite Bio‐Technology Co., Ltd (purity > 98.0%, HPLC; Nanjing, China). H_2_O_2_ solution (30%), N‐acetyl‐L‐cysteine (NAC), Drp1 small‐interfering RNA (siRNA), and scramble control were purchased from Santa Cruz Biotechnology.

### 2.2 Cell culture and lentivirus gene transfer

Conditionally immortalized mouse podocytes (MPC5) were purchased from Rantai Company (Shanghai, China). MPC5 cells were cultured according to the previously described method (Mundel, Reiser, & Kriz, 1997 ). In brief, podocytes were cultured in collagen I‐coated dishes (BD Biosciences, Bedford, MA) in RPMI 1640 (Invitrogen, Carlsbad, CA) supplemented with 10% FBS (Gibco BRL, Grand Island, NY) 100 U/ml penicillin G, 0.1 mg/ml streptomycin (Gibco BRL), and 50 IU/ml of recombinant murine IFN‐γ (Invitrogen, Carlsbad, CA) at 37°C and 5% CO_2_. To induce differentiation, cells were treated for 12–14 days at 37°C, and the medium was replaced with RPMI 1640 (containing 5% FBS without IFN‐γ). For the experiments, the medium was replaced with Dulbecco's modified Eagle's medium (DMEM), supplemented with 1% FBS and 5.3 mM D‐glucose (Invitrogen). The differentiated MPC5 cells were treated with 5.3 mM D‐glucose (NG group), 30 mM D‐glucose (HG group); 30 mM D‐glucose + 25 mM PD (HG + PD group); or 5.3 mM D‐glucose + 25 mM PD (NG + PD group) for 24, 48, and 72 hrs. The lentiviruses were added to the cells together with Polybrene (Sigma–Aldrich) at a final concentration of 10 mM for 6 hr (at 37°C) and cultured in F‐12 Ham's medium.

### 2.3 Animals and experimental protocols

All animal study protocols for the present study were reviewed and approved by the Institutional Animal Care and Use Committee at Nanfang Medical University, China. In brief, KKAy mice and their nondiabetic control C57BL/6Jmice (9–11 weeks of age) were purchased from the Institute of Laboratory Animal Sciences, Chinese Academy of Medical Science (Beijing, China). Mice were individually housed in stainless steel cages in a room at a constant temperature and humidity with 12 hr light/dark cycles. During the experiment, the KKAy mice were provided high‐fat diet and water ad libitum. Male C57BL/6J mice were pair‐fed with normal diet and water ad libitum. At 14 weeks of age, urine samples were collected, and KKAy mice with a urine albumin‐creatinine ratio (ACR) ≥300 µg/mg were considered to have DN. The KKAy mice were randomly divided into a PD treatment group (DIAB + PD, *n* = 10), which received 100 mg/kg PD for 8 weeks, or a DN control group (DIAB, *n* = 10), which was administered the same volume of vehicle (containing 0.5% sodium carboxymethylcellulose). Age‐matched healthy C57BL/6J mice receiving vehicle were used as a normal control group (CTRL, *n* = 10), and mice receiving 100 mg/kg of PD for 8 weeks served as the PD control group (CTRL + PD, *n* = 10). At the end of the study, urine was collected from individual mice housed in metabolic cages for 24 hr. All mice were sacrificed by carbon dioxide asphyxiation. Blood samples were collected, and plasma and serum were separated and stored at −20°C until final biochemical analysis. Kidneys samples were fixed in 10% buffered formalin and frozen at −80°C until use.

### 2.4 Acute toxicology test

The oral median lethal dose (LD50) was determined in C57BL/6J mice using an “up‐and‐down OECD procedure” (Buschmann, 2013 ). Briefly, 10 mice were orally administered a fixed dose of PD (5000 mg/kg body weight) after fasting overnight, and any signs and symptoms of toxicity and mortality were recorded, if any, up to a period of 72 hr.

### 2.5 Biochemical analysis

A total of 24 hr urine protein (UP), urinary albumin excretion (UAE), blood urea nitrogen (BUN), and serum creatinine (SCr) levels were analyzed by the Department of Clinical Laboratory at the Shenzhen Hospital of Nanfang Medical University. Renal hypertrophy was assessed using the kidney weight to body weight ratio (KW/BW).

### 2.6 Isolation of kidney glomeruli and mitochondria

Glomeruli were isolated using a previously described method (Takemoto et al., 2002 ) using a kit purchased from Sigma (Sigma–Aldrich) according to the manufacturer's protocol. Briefly, mice were anesthetized and the kidney was removed and perfused with 5 ml of phosphate‐buffered saline, then kidneys were minced into small pieces, digested by collagenase and DNase, and filtered. After washing for three times, the glomeruli were collected using a magnet and the purity of glomeruli was confirmed to be about 95% by phase‐contrast microscopy. Mitochondria from glomeruli were isolated using a kit purchased from Sigma, according to the manufacturer's protocol. Isolated mitochondria were resuspended in radioimmunoprecipitation assay buffer at 4°C and then centrifuged at 12,000*g* for 10 min at 4°C. The protein concentration was determined using the Bradford method.

### 2.7 Histopathological analysis

Mice kidneys were excised, fixed in 4% paraformaldehyde, dehydrated, and embedded in paraffin, sectioned at 3 µm, and stained with hematoxylin and eosin.

### 2.8 Western blot analyses

MPC5 cells or isolated glomeruli were homogenized in an ice‐cold lysis buffer (20 mM tris‐HCl, pH 7.5, 150 mM NaCl, 1 mM Na2EDTA, 1 mM EGTA, 1% Triton X–100, 1 mM PMSF, 200 mM sodium fluoride, 4 mM sodium orthovanadate as protease inhibitors) for 20 min. The Bio–Rad protein assay was used to test the protein concentration. The following antibodies were used: anti‐Drp1 antibody (1:200), anti‐p(616)‐Drp1 (1:200), anti‐caspase3 antibody (1:500), anti‐nephrin antibody (1:200), anti‐podocin antibody (1:500), and anti‐cytochrome C antibody (1:1000). Anti‐β‐actin antibody (1:1000) from Sigma (Sigma–Aldrich) was used as a loading control (Pierce, Rockford, IL).

### 2.9 Reverse‐transcriptase quantitative PCR (RT‐qPCR) analysis

Total DNA and RNA from cultured renal cortices and MPC5 cells were extracted using Trizol Reagent (Gibco Life Technologies). The cDNA was then quantified by real‐time PCR (Applied Biosystems, Foster City, CA), a SYBR Green PCR Mix Kit (TOYOBO, Osaka, Japan), and appropriate primers. Primers were purchased from Xibao Biotech Co., Ltd. (Shanghai, China); primer information is presented in Table 1 . The cycling conditions were 10 min at 95°C, followed by 40 cycles of 95°C for 15 s, 40 s at 60°C, and 1 min at 73°C.

**Table 1:** Primer sequences for RT‐PCR

Gene symbol	Accession number	Forward primer (50−30)	Reverse primer (50−30)
Nephrin	NM 149329	TTCAGACCACACCAACATCC	AGCCAGGTTTCCACTCCA
Podocin	NM 1571216	GTGAGGAGGGCACGGAAG	AGGGAGGCGAGGACAAGA
GAPDH	NM 132914	GTCTTCACTACCATGGAGAAGG	TCATGGATGACCTTGGCCAG

GAPDH, glyceraldehyde 3‐phosphate dehydrogenase.

### 2.10 Evaluation of podocyte apoptosis in renal tissue

Podocyte apoptosis was evaluated using a commercially available terminal deoxynucleotidyl transferase‐mediated dUTP nick‐end labeling (TUNEL) assay kit (CHEMICON International, Temecula, CA) according to the manufacturer's protocol. The sections of frozen kidney tissue were counterstained with DAPI, anti‐synaptopodin antibody, and TUNEL. TUNEL‐positive cells (Green) were analyzed via fluorescence microscopy (Nikon Corporation, Tokyo, Japan).

### 2.11 Hoechst 33258 staining

Podocytes were fixed with 4% formaldehyde in phosphate‐buffered saline (PBS) for 10 min, and the cells were stained with Hoechst 33258 for 1 hr. Apoptotic cells were identified by the condensation and fragmentation of their nuclei, and images were captured using a fluorescence microscope (Nikon Corporation, Tokyo, Japan).

### 2.12 Analysis of mitochondrial cytochrome C release

MPC5 cells were cultured for 72 hr in 5.3 mM or 30 mM D‐glucose, harvested by gentle scraping into the medium, and washed with ice‐cold phosphate‐buffered saline (PBS). Mitochondrial fractions containing cytochrome C were obtained by using a Mitochondria Isolation kit (Sigma–Aldrich). The pellet contained the mitochondrial fraction, and the supernatant was collected as the cytosolic fraction. The release of cytochrome C from the mitochondria into the cytoplasm was detected by Western blot quantification.

### 2.13 Measurement of caspase‐3 and ‐9 activities in podocytes

The activities of caspase‐3 and ‐9 were measured using a commercially available colorimetric protease Apotag assay kit (Invitrogen) through cleavage of the substrate (Ac‐DEVD‐pNA forcaspase‐3, Ac‐LEHD‐ pNA for caspase‐9) and release of a (pNA) chromophore. The activities of caspase‐3 and ‐9 are expressed as the absorbance of the cleaved substrate (pNA) at 405 nm.

### 2.14 Transmission electron microscopy (TEM)

Podocytes or renal cortex were fixed in 2.5% glutaraldehyde and then fixed in 1% O_s_O_4_/0.1 mol/L phosphate buffer (Sigma–Aldrich) for 1 hr. Ultrathin sections, 50 nm thick, were cut with an Ultracut S Ultramicrotome, placed on copper grids, double‐stained with uranyl acetate and lead citrate, and examined by TEM (LSM 510; Carl Zeiss, Germany). The ultrastructural changes to the podocyte slit diaphragm of the mice (Ruotsalainen et al., 1999 ) were measured as previously described.

### 2.15 Mitochondrial membrane potential (MMP) by florescent  JC1

The MMP in the podocytes and isolated mitochondria were determined using JC‐1 fluorescent staining according to manufacturer's protocol as described previously (Daehn et al., 2014 ). The isolated mitochondria and cells were washed twice with ice‐cold Hank's balanced salt solution (HBSS) (Gibco BRL) centrifuged at 400 × *g* for 1 min at 4°C. Cells or mitochondria were washed with PBS twice; the results were read using a microplate reader (FLUOStar Omega, BMG Labtech, Ortenberg, Germany). The ratio was calculated by relative aggregate fluorescence (red) to JC‐1 monomer (green).

### 2.16 ATP production

After incubation for 3 days in the presence or absence of 25 mM PD in F‐12 Ham's medium containing 5.3 mM or 30 mM D‐glucose at 37°C, MPC5 cells or isolated glomeruli were incubated for 1 hr in medium with 5.3 mM or 30 mM D‐glucose at 37°C. ATP was extracted in 0.1% trichloroacetic acid and neutralized in 0.1 mol/L Tris acetate. ATP levels of podocytes and isolated glomeruli were determined using CellTitre‐Glo Luminescent Cell Viability Assay Kit (Beyotime, Jiangsu, China). ATP assays were performed as previously described, and ATP levels were measured and normalized to total protein concentration.

### 2.17 Mitochondrial ROS determination

Intracellular ROS generation was detected as described previously (Zhang, Wang, & Chen, 2012 ). Isolated glomeruli and MPC5 cells were stimulated with 30 mM D‐glucose or 25 mM PD for 3 days. The collected cells were washed twice with wash buffer and were directly analyzed using a fluorescence microplate reader. More than 10,000 cells were acquired and analyzed for each sample, and the ROS generation was normalized to the protein concentration of each treated sample relative to control. Fluorescence was monitored by a plate reader fluorometer (Molecular Devices, Sunnyvale, CA).

## 3 RESULTS

### 3.1 The acute toxicology testing of PD

The acute toxicology of PD was evaluated in mice by orally administrating the mice with a single large dose of PD at 5000 mg/kg body weight. The mice were subsequently observed for 72 hr. No behavioral changes or death were observed.

### 3.2 PD improves renal function in KKAy mice

To investigate the effect of PD in KKAy mice kidneys during DN, we administered vehicle or 100 mg/kg/day PD orally to KKAy and C57BL/6J mice for 8 weeks. The KW/BW ratio, 24 hr UP, UAE, SCr, and BUN levels significantly increased in the DIAB mice when compared with CTRL mice. The oral administration of PD effectively decreased the fasting blood glucose (FBG), KW/BW ratio, 24 hr UP, UAE, SCr, and BUN levels relative to DIAB mice (Table 2 ). However, PD treatment did not affect the FBG levels or renal function of CTRL mice.

**Table 2:** Effects of PD on the biological parameters of the KKAy mice

Biochemical parameter	CTRL	DIAB	CTRL + PD	DIAB + PD
FBG (mM)	5.8 ± 0.2	23.7 ± 1.3^*^	5.7 ± 0.3^*^	7.4 ± 0.2^*^ ^,^ ^**^
Kidney weight/body weight (×10^−3^)	0.6 ± 0.1	1.4 ± 0.1^*^	0.5 ± 0.2^*^	0.8 ± 0.1^*^
24 hr urinary protein (mg/24 hr)	7.1 ± 0.4	29.7 ± 0.8^*^	6.9 ± 0.4^**^	8.0 ± 0.7^**^
Urinary albumin (mg/24 hr)	0.31 ± 0.03	1.01 ± 0.03^*^	0.33 ± 0.02^**^	0.45 ± 0.02^*^ ^,^ ^**^
Serum creatinine (μM)	42.7 ± 1.2	98.2 ± 2.1^*^	44.1 ± 0.9^**^	52.1 ± 0.8^**^ ^,^ ^**^
Blood urea nitrogen (mmol/L)	5.7 ± 0.4	19.6 ± 0.2^*^	5.8 ± 0.3^**^	6.4 ± 0.6^**^

Effects of PD on FBG, kidney weight/body weight ratio, 24‐UP, UAE, SCr, and BUN levels in KKAy mice. Mice were orally treated with PD or vehicle for 8 weeks. CTRL, C57BL/6J mice treated with vehicle; DIAB, KKAy mice treated with vehicle; DIAB + PD, KKAy mice treated with PD; CTRL + PD group, C57BL/6J mice treated with PD. Data are expressed as the mean ± S.E. (*n* = 10).

^*^ *p* < 0.05 versus CTRL.

^**^ *p* < 0.05 versus DIAB.

### 3.3 PD attenuates podocyte apoptosis in KKAy mice

**Figure 1:**
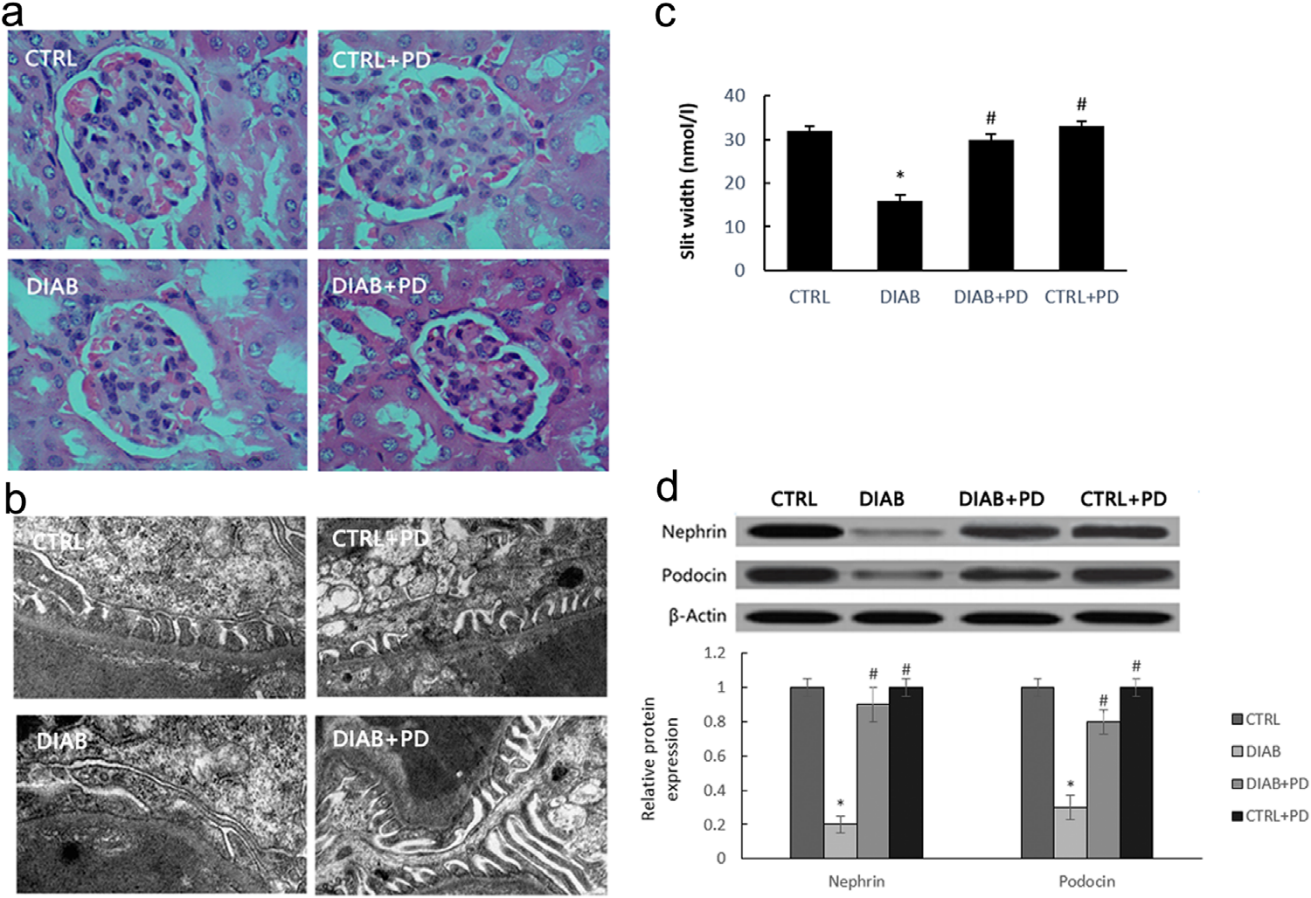
PD attenuates podocyte apoptosis in KKAy mice. Male C57BL/6J and KKAy mice were administered PD (100/kg/day) for 8 weeks, (a) Kidney histology (×200). (b) TEM (×25,000) of renal tissue from KKAy mice. (c) The quantitative analysis of the slit width. (d) The influence of PD treatment on protein levels of nephrin and podocin. CTRL, C57BL/6J mice treated with vehicle; DIAB, KKAy mice treated with vehicle; DIAB + PD, KKAy mice treated with PD; CTRL + PD, C57BL/6J mice treated with PD. Data are presented as the mean ± SE (*n* = 10). *p < 0.05 versus CTRL; #*p* < 0.05 versus DIAB

In order to study the effect of PD on podocyte in diabetic milieu. We studied KKAy mice that had been treated with PD for 8 weeks. The kidneys of the mice were examined histopathologically. KKAy mice exhibited significant mesangial expansion compared with C57BL/6J mice, and PD treatment prevented these changes and restored normal structure to the glomeruli (Figure 1 a). In the kidneys of KKAy mice, the foot processes exhibited extensive fusion and filtration slits, and pore density was reduced. By contrast, KKAy mice treated with PD exhibited a restoration of nephrin and podocin protein levels and a normalization of the shape of the foot processes and podocyte slit pores (Figure 1 b–d). By contrast, there were no changes in the histologic or biochemical characteristics of C57BL/6J mice in the presence or absence of PD treatment.

### 3.4 PD attenuates apoptosis in hyperglycemic MPC5 cells

**Figure 2:**
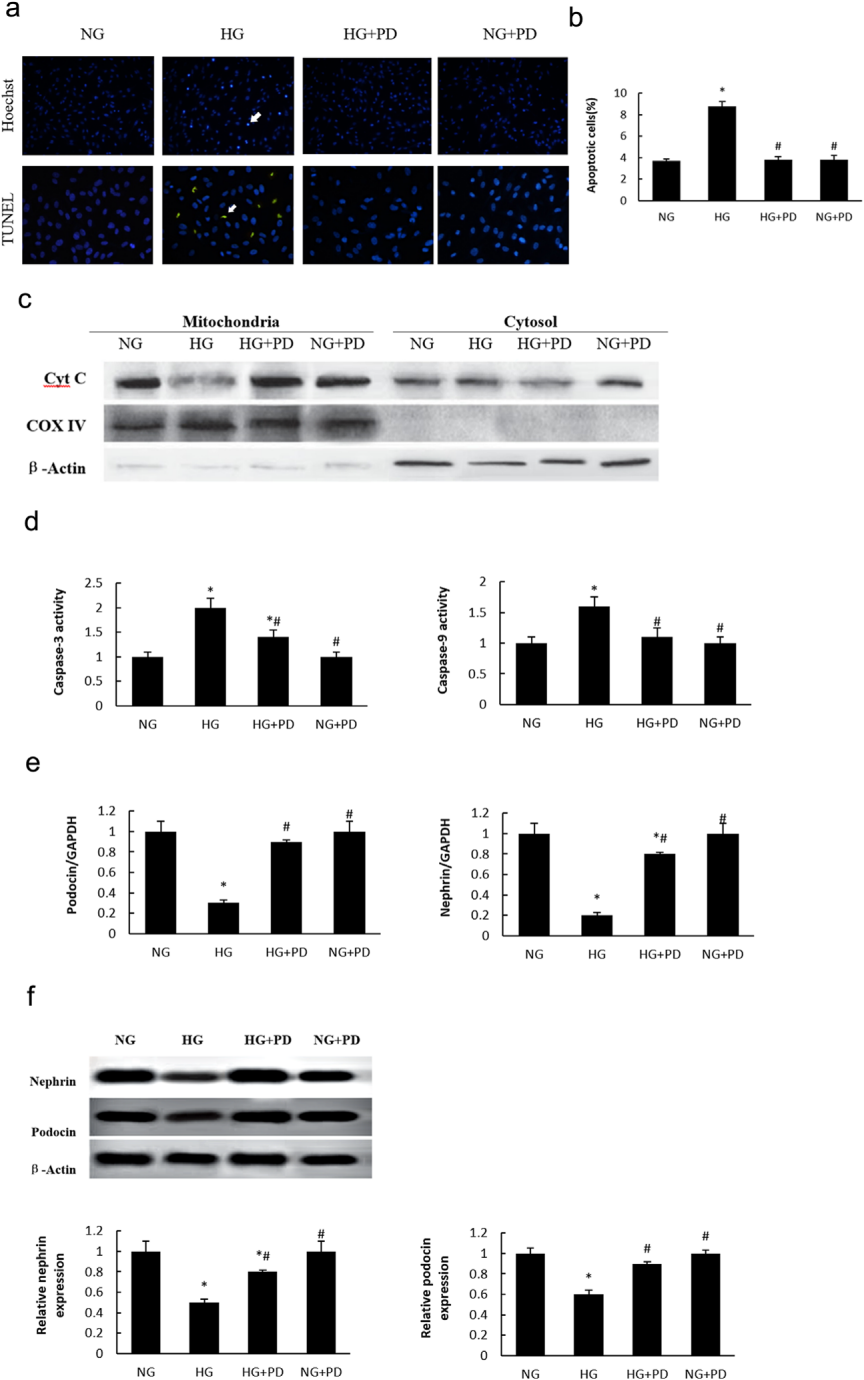
PD attenuated apoptosis on HG‐induced MPC5 cell. (a) Apoptosis measured by Hoechst 33258 staining and TUNEL staining (×200). (b) The quantitative determination of cell apoptosis using flow cytometry. (c) Western blot analysis of cytochrome C release. (d) Cleaved form of caspase‐3 and caspase‐9. (e) Changes in nephrin and podocin mRNA expression levels in hyperglycemic MPC5 cells. (f) Western blot analysis of nephrin and podocin. NG, MPC5 cells cultured in 5.3 mM glucose; HG, MPC5 cells cultured in 30 mM D‐glucose; HG + PD, MPC5 cells cultured in 30 mM glucose and 25 mM PD; NG + PD, MPC5 cells cultured in 5.3 mM glucose and 25 mM PD. The results are presented as the mean ± SE (*n* = 4; ≥100 cells were counted in three independent experiments). *p < 0.05 versus NG; #*p *< 0.05 versus HG

As shown in Figure 2 , podocyte apoptosis was measured using the TUNEL assay, Hoechst 33258 staining, and flow cytometry. Treatment with PD significantly prevented the decrease of cell viability. We further investigated the effects of PD on cytochrome C release and the activation of the caspase cascade. We observed a significant release of cytochrome C from the mitochondria after exposure to HG, whereas PD treatment blocked cytochrome C release (Figure 2 c). HG activated caspase‐3 (cleaved form) and ‐9, which suggested that HG‐induced podocyte apoptosis was mediated through the intrinsic mitochondrial proapoptotic pathway, and that the activation of both caspase‐3 and ‐9 were substantially reduced by PD (Figure 2 d). Furthermore, real‐time PCR and Western blot analysis revealed that HG‐induced reduction of podocin and nephrin was restored by PD treatment (Figure 2 e,f).

### 3.5 PD inhibits HG‐induced mitochondria fission

**Figure 3:**
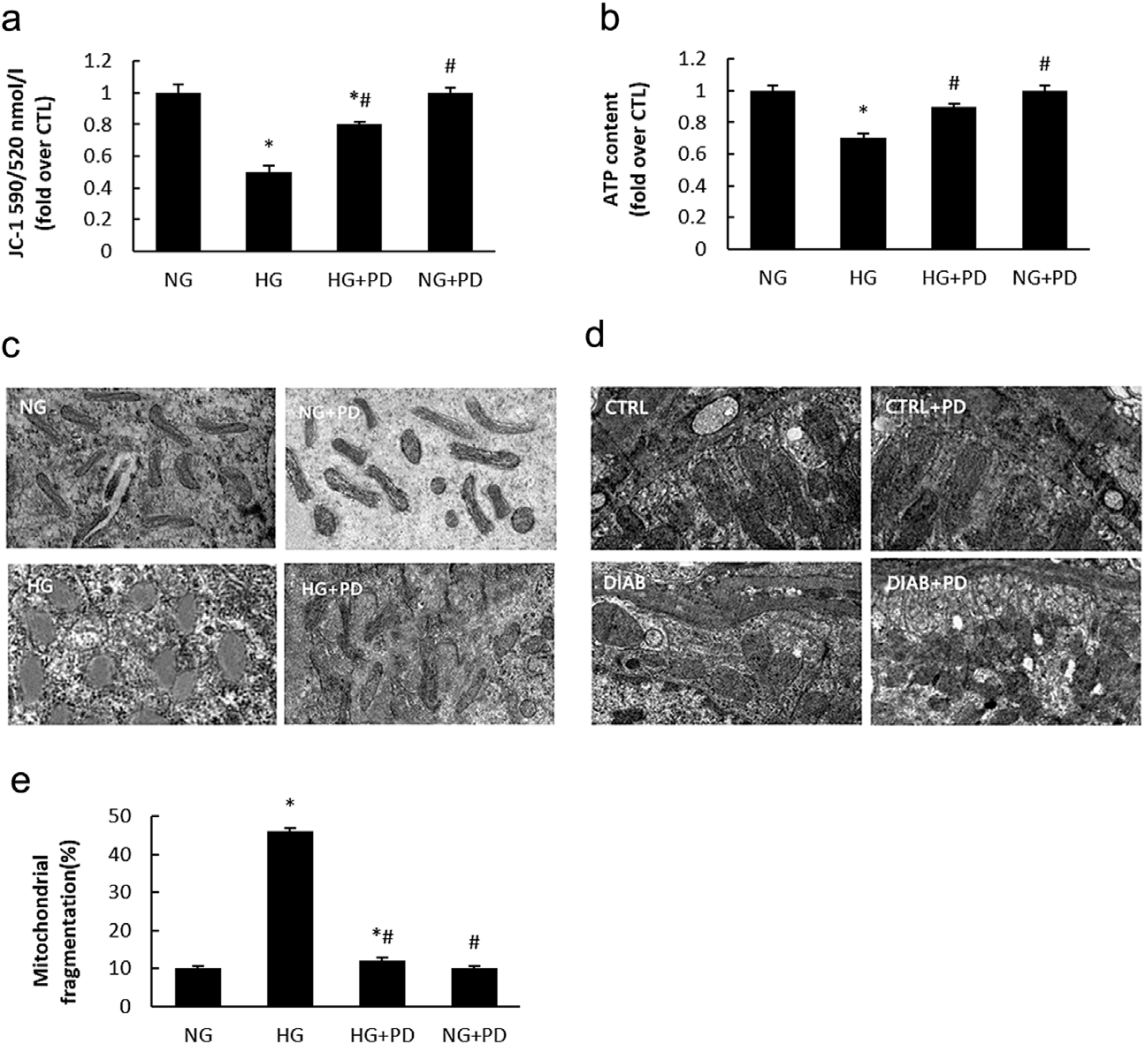
PD inhibits HG‐induced mitochondria fission. (a) Changes in MMP expression levels were measured by flow cytometry using JC1. (b) Adenosine triphosphate (ATP) production. (c) Transmission electron microscopy images of the mitochondria in MPC5 cells (×25,000). (d) Mitochondrial morphology in glomerular podocytes from mice (×25,000). (e) The quantitative determination of mitochondrial fragmentation in MPC5 cells. NG, MPC5 cells cultured in 5.3 mM D‐glucose; HG, MPC5 cells treated with 30 mM D‐glucose; HG + PD, MPC5 cells cultured in 30 mM D‐glucose and 25 mM PD; CTRL + PD, MPC5 cells cultured in 5.3 mM D‐glucose, and 2 5 mM PD. CTRL, C57BL/6J mice treated with vehicle; DIAB, KKAy mice treated with vehicle; DIAB + PD, KKAy mice treated with PD; CTRL + PD, C57BL/6J mice treated with PD. The results are presented as the mean ± SE. *p < 0.05 versus CTRL(NG); #*p* < 0.05 versus HG(DIAB)

Compared with the mitochondria in NG cells, the mitochondria in hyperglycemic MPC5 cells exhibited a more punctate pattern. However, this fragmentation could be prevented by PD treatment. We also analyzed MMP (Figure 3 a) and ATP production (Figure 3 b) in hyperglycemic MPC5 cells after PD treatment. Subsequently, we investigated the mechanism underlying the reversion effect of PD on podocyte mitochondria. As shown in Figure 3 c,d, the ultrastructural electron microscopy analysis revealed marked differences in the shape of the mitochondria in HG cells. After PD treatment, the mitochondria maintained a long, filamentous structure. The podocyte mitochondria from KKAy mice were notably swollen, deformed, and vesicular compared with those from C57BL/6J mice. By contrast, KKAy mice treated with PD exhibited relatively few swollen, deformed, and vesicular mitochondria (Figure 3 e).

### 3.6 PD blocks Drp1 expression in hyperglycemic podocytes

**Figure 4:**
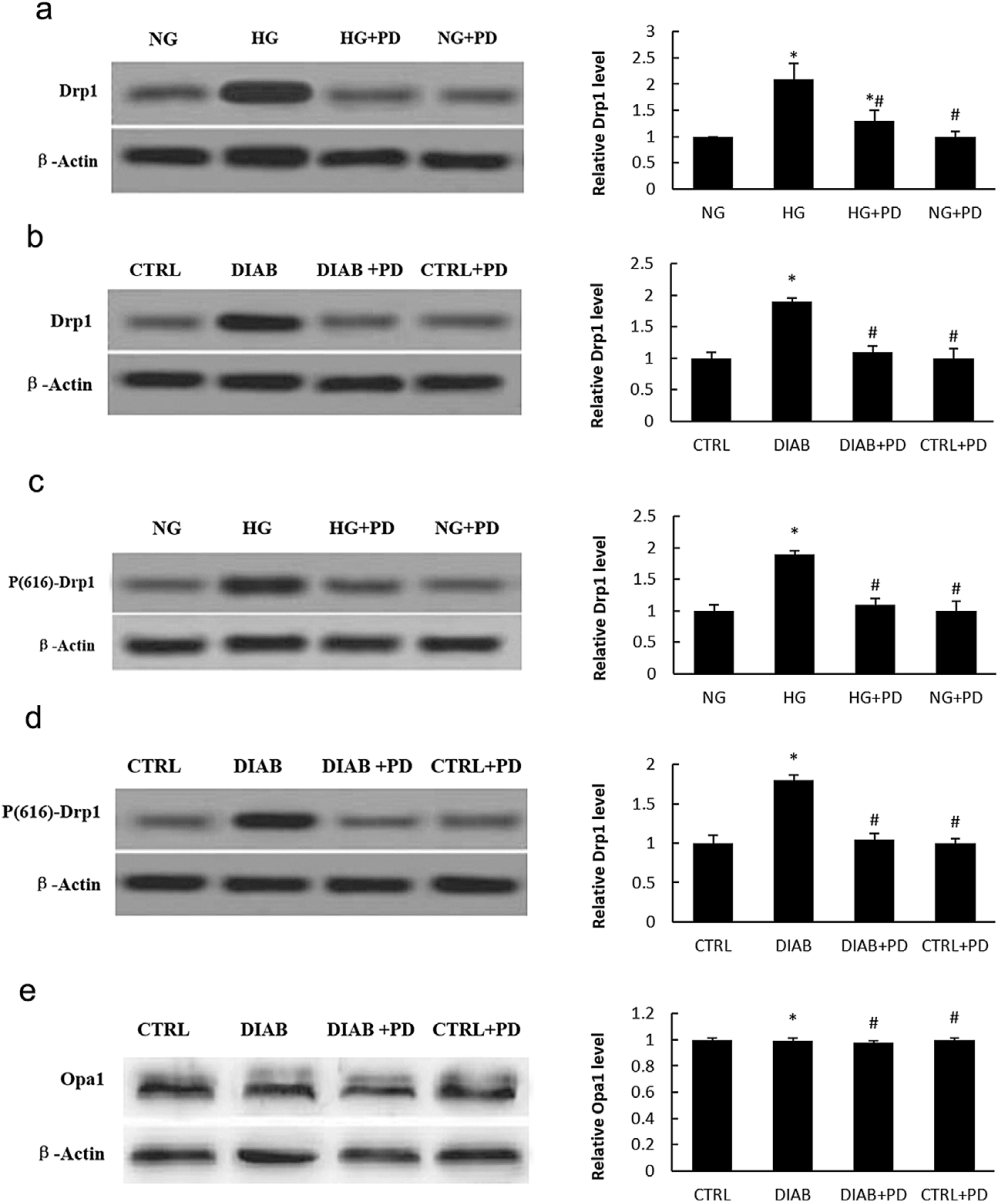
PD inhibits Drp1 expression in HG‐induced podocytes. (a) Drp1 expression in MPC5 cells. (b) Drp1 expression in primary podocytes isolated from KKAy mice. (c) Active Drp1 (p‐Drp1) levels in MPC5 cells. (d) Active Drp1 (p‐Drp1) levels in primary podocytes isolated from KKAy mice. (e) Western blot analysis of Opa1 expression in isolated glomerular podocytes. NG, MPC5 cells cultured in 5.3 mM D‐glucose; HG, MPC5 cells cultured in 30 mM D‐glucose; HG + PD, MPC5 cells cultured in 30 mM D‐glucose and 25 mM PD; CTRL + PD, MPC5 cells cultured in 5.3 mM D‐glucose and 25 mM PD. CTRL, C57BL/6J mice treated with vehicle; DIAB, KKAy mice treated with vehicle; DIAB + PD, KKAy mice treated with PD; CTRL + PD, C57BL/6J mice treated with PD. The results are presented as the mean ± SE. *p < 0.05 versus CTRL(NG); #*p* < 0.05 versus HG(DIAB)

Drp1‐mediated fission is involved in mitochondrial fragmentation (Filichia, Hoffer, Qi, & Luo, 2016 ) and acts with other cofactors to induce cytochrome C release, caspase activation, and apoptosis (Oettinghaus et al., 2016 ). Hence, we investigated whether HG or PD could affect the expression of Drp1 in podocyte mitochondria. We isolated and cultured primary podocytes from KKAy mice. As shown in Figure 4 , HG strongly increased Drp1 expression in both MPC5 cells (Figure 4 a) and primary podocytes (Figure 4 b) from KKAy mice. However, treatment of PD effectively abolished the elevated protein expression level of Drp1 induced by HG. We then examined phosphorylated Drp1 (p‐Drp1) at serine residue 616 in MPC5 cells (Figure 4 c) and primary podocytes (Figure 4 d). p‐Drp1 was significantly increased after HG treatment, and the increase in the levels of this active form of Drp1 was blocked by PD.

There are several key molecules that modulate mitochondria morphology. In addition to Drp1, which mediates mitochondria fission under normal conditions, Opa1, and mitofusin (Atkins, Dasgupta, Chen, Mewburn, & Archer, 2016 ) are required to promote mitochondria fusion. Many studies have clearly demonstrated that loss of Opa1 induces cell mitochondrial fragmentation and apoptosis (Kushnareva et al., 2013 ). We thus analyzed the Opa1 levels in primary podocytes and determined that the level of Opa1 was not affected by HG or PD (Figure 4 e).

### 3.7 PD inhibits mitochondrial fission and cell apoptosis in podocytes by suppressing Drp1 expression

**Figure 5:**
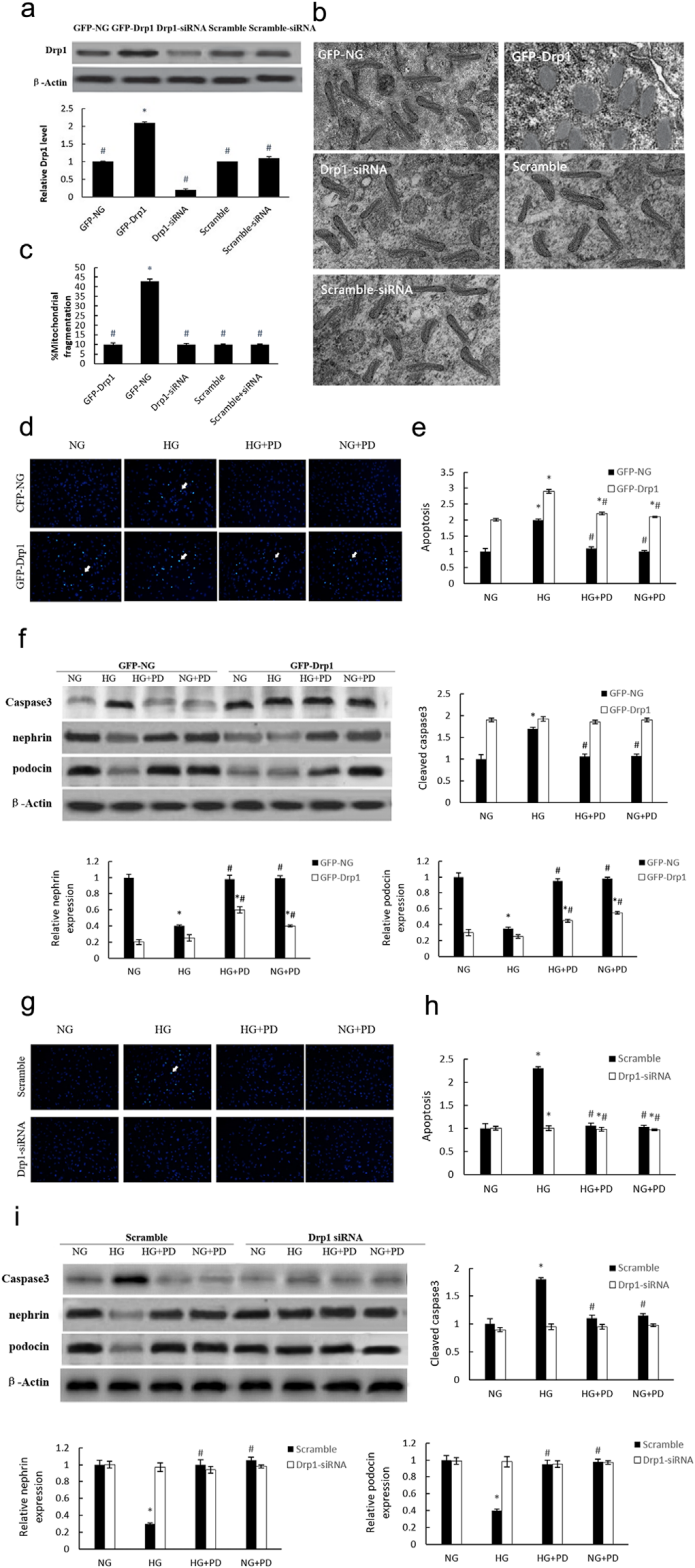
The influence of Drp1 on mitochondrial fission and cell apoptosis in MPC5 cells. (a) Drp1 expression in GFP‐Drp1‐ or siRNA‐infected MPC5 cells. (b) Transmission electron microscopeimages of the mitochondria in MPC5 cells (×25,000). (c) Mitochondrial morphology in GFP‐Drp1‐infected MPC5 cells (×2500). (d) Apoptosis of GFP‐Drp1‐infected MPC5 cells measured by Hoechst 33258 staining (×200). (e) The quantitative determination of cellular apoptosis in GFP‐Drp1‐infected MPC5 cells using flow cytometry. (f) Western blot analysis for caspase‐3 (cleaved form), nephrin and podocin expression in GFP‐Drp1‐infected MPC5 cells. (g) Apoptosis of Drp1 siRNA‐infected MPC5 cells measured by Hoechst 33258 staining (×200). (h) The quantitative determination of cellular apoptosis in Drp1 siRNA‐infected MPC5 cells using flow cytometry. (i) Western blot analysis for caspase‐3 (cleaved form), nephrin, and podocin expression in Drp1 siRNA‐infected MPC5 cells. GFP‐NG, MPC5 cells infected with empty lentivirus; GFP‐Drp1, MPC5 cells infected with lentivirus expressing Drp1; Drp1 siRNA, MPC5 cells infected with Drp1 siRNA; Scramble, MPC5 cells infected with lentivirus expressing scramble oligonucleotide; Scramble siRNA, MPC5 cells infected with lentivirus expressing scramble siRNA; HG, MPC5 cells cultured in 30 mM D‐glucose; HG + PD, MPC5 cells cultured in 30 mM glucose and 25 mM PD; NG + PD, MPC5 cells cultured in 5.3 mM glucose and 25 mM PD. The results are presented as the means ± SE. *p < 0.05 versus NG(GFP‐NG); #*p* < 0.05 versus HG(GFP‐Drp1)

We further examined the mechanism that links PD and Drp1. The level of Drp1 expression and mitochondrial fragmentation in podocytes were significantly elevated or reduced when cells were infected with a Drp1‐GFP lentivirus or a Drp1 siRNA lentivirus, respectively. The relative levels of Drp1 and mitochondria fragmentation suggested that Drp1 plays a key role in mitochondrial fission (Figure 5 a–c). Furthermore, we observed increased apoptosis and caspase‐3 activity in podocytes infected with the Drp1‐GFP lentivirus (Figure 5 d–f). Moreover, podocin and nephrin protein concentrations were also decreased (Figure 5 g), and the reversion effect of PD on HG‐induced cell apoptosis was largely abolished by Drp1 overexpression. In contrast to Drp1 activation, the downregulation of Drp1 reversed podocytes apoptosis induced by HG. (Figure 5 h–i). Therefore, we concluded that the reversion effect of PD in HG‐induced podocyte dysfunction might be due to the suppression of Drp1 expression.

PD decrease HG−induced Drp1 expression via suppressing cellular ROS production.

PD decreases HG−induced Drp1 expression by suppressing cellular ROS production.

**Figure 6:**
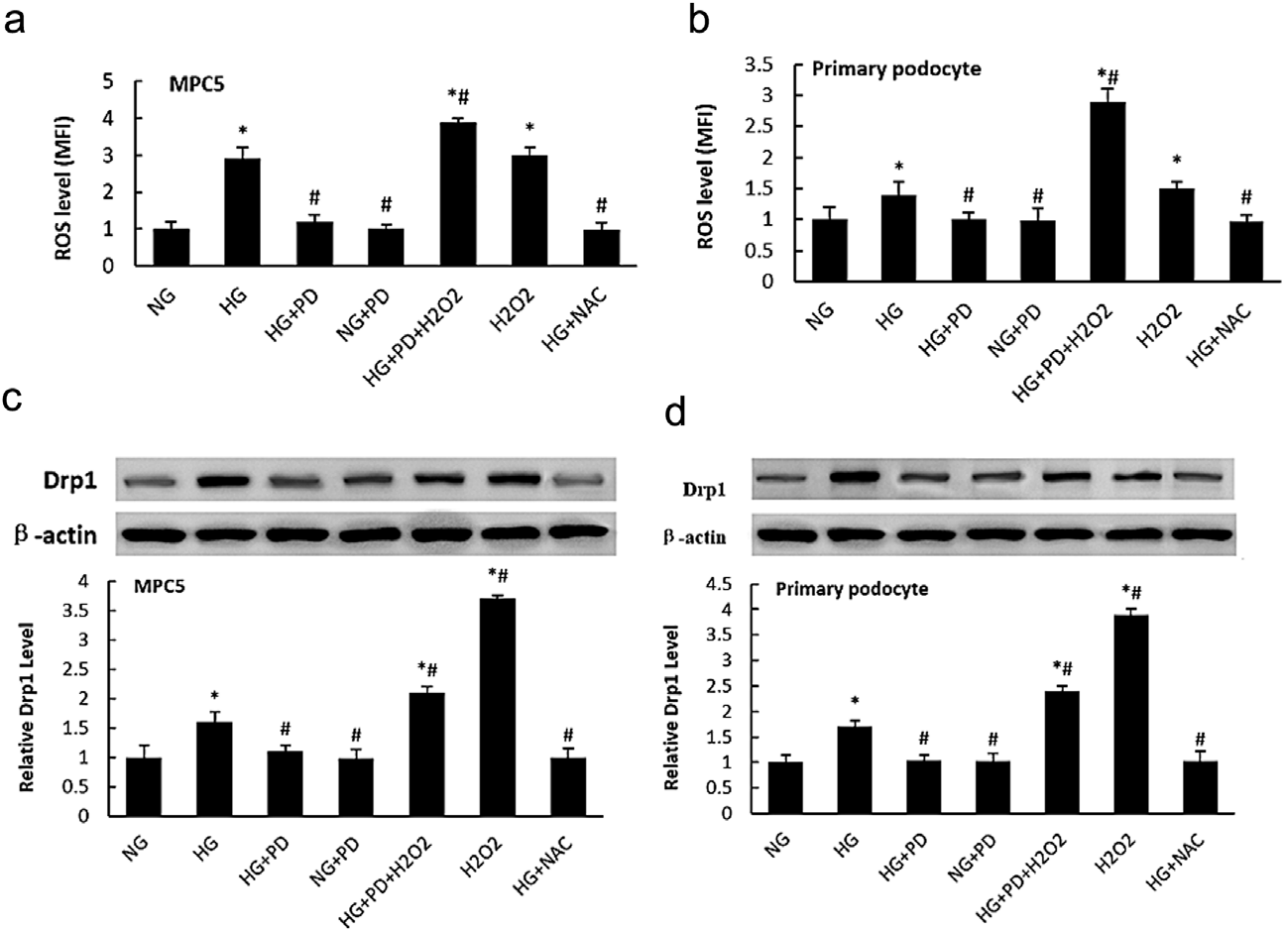
PD attenuated ROS production in HG‐induced podocyte. (a) Flow cytometry analysis of ROS levels in MPC5 cells. (b) Flow cytometry analysis of ROS levels in primary podocytes from KKAy mice. (c) Western blot analysis for Drp1 levels in MPC5 cells. (d) Western blot analysis for Drp1 levels in primary KKAy podocytes. NG, MPC5 cells (primary podocytes) cultured in 5.3 mM D‐glucose; HG, MPC5 cells (primary podocytes) cultured in 30 mM D‐glucose; HG + PD, MPC5 cells (primary podocytes) cultured in 30 mM D‐glucose and 25 mM PD; NG + PD, MPC5 cells (primary podocytes) cultured in 5.3 mM D‐glucose and 25 mM PD; HG + PD + H2O_2_, MPC5 cells (primary podocytes) treated with 30 mM D‐glucose, 25 mM PD and 1 µmol/L H_2_O_2_; H_2_O_2_, MPC5 cells (primary podocytes) cultured in 5.3 mM D‐glucose and 1 µmol/L H_2_O_2_; HG + NAC, MPC5 cells (primary podocytes) cultured in 30 mM D‐glucose and 0.4 mmol/L NAC. The results are presented as the mean ± SE. *p < 0.05 versus NG; #*p* < 0.05 versus HG

Cellular ROS play crucial roles in the initiation of cell apoptosis and mitochondrial dysfunction (Bukowska et al., 2008 ). To investigate the effect of PD on ROS generation in hyperglycemic podocytes, MPC5 cells or isolated mice podocytes were cultured with or without (25 mM) in normal‐glucose (5.3 mM D‐glucose, CTRL group), and high‐glucose media (30 mM D‐glucose, HG group). The intracellular level of ROS was markedly higher in the HG group compared with the NG group (Figure 6 ). However, PD treatment nearly completely abolished HG‐induced ROS production in MPC5 cells and isolated mice podocytes. As shown in Figure 6 c,d, the protein expression level of Drp1 and ROS production were both significantly increased in the HG group compared with NG, and these increases were blocked by PD in both MPC5 cells and primary podocytes. Furthermore, the production of ROS and the expression of Drp1 were strongly reduced or enhanced by the ROS scavenger NAC (0.4 mmol/L) or a pro‐oxidant agent H2O2 (1 µmol/L) respectively (Figure 6 a–d) under HG, respectively. PD and NAC significantly reduced the expression of Drp1 in hyperglycemic MPC5 cells and primary podocytes, which suggests that the induction of Drp1 under HG may be at least partially associated with the release of ROS.

### 3.8 PD prevents cell apoptosis and ROS production in Drp1 siRNA‐infected primary podocytes

**Figure 7:**
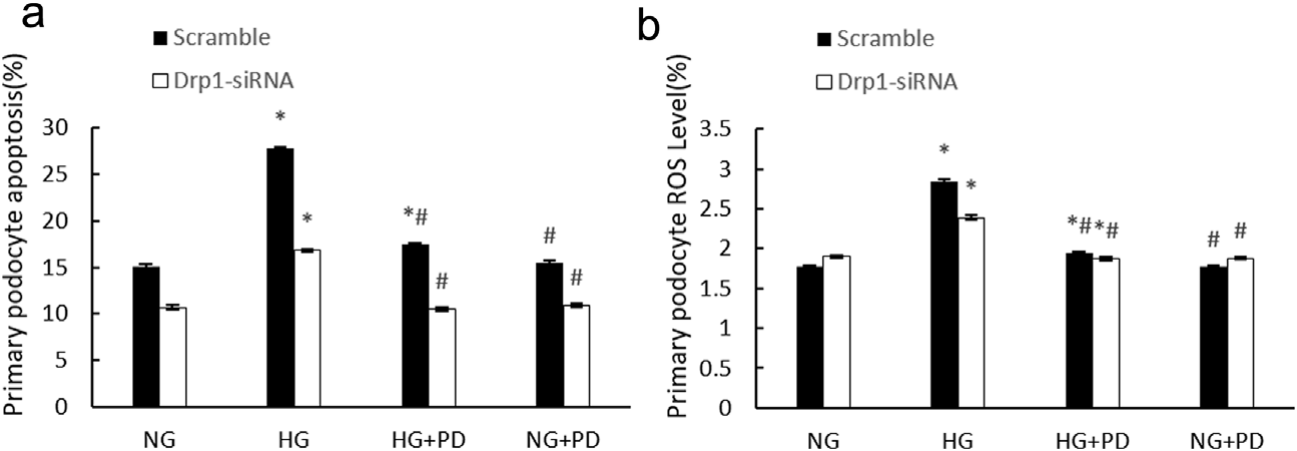
PD inhibits cell apoptosis and ROS production in Drp1 siRNA‐infected primary podocytes. (a) Quantitative determination of cell apoptosis in Drp1 siRNA‐infected primary podocytes using flow cytometry. (b) Flow cytometry analysis of ROS levels in Drp1 siRNA‐infected primary podocytes. NG, primary podocytes (Scramble or siRNA infected) cultured in 5.3 mM D‐glucose; HG, primary podocytes (Scramble or siRNA infected) cultured in 30 mM D‐glucose; HG + PD, primary podocytes (Scramble or siRNA infected) cultured in 30 mM D‐glucose and 25 mM PD; NG + PD, primary podocytes (Scramble or siRNA infected) cultured in 5.3 mM D‐glucose and 25 mM PD. The results are presented as the mean ± SE (*n* = 10).*p < 0.05 versus NG; #*p* < 0.05 versus HG

As shown in Figure 7 , primary podocytes treated with HG + Drp1‐siRNA + PD exhibited decreased apoptosis compared with cells treated with HG + Drp1‐siRNA, and ROS levels in primary podocytes were affected by HG but not Drp1‐siRNA.

## 4 DISCUSSION

Murine models of diabetes mellitus suggest that podocyte apoptosis is a key mediator in the pathogenesis of DN (Zhou et al., 2012 ). In the present study, we demonstrated that PD can attenuate HG‐induced podocyte apoptosis by inhibiting ROS production and subsequent down‐regulation of Drp1 expression.

PD is an active stilbene compound isolated from the roots of Polygonumcuspidatum. PD has been manifested to possess antioxidative and anti‐inflammatory activities (Mohan et al., 2013 ). PD possesses antioxidative and anti‐inflammatory activities (Mohan et al., 2013 ). Previous studies have demonstrated the therapeutic effects of PD in HG‐induced injury in multiple organs, including heart, liver, and brain (Xu et al., 2016 ; Zhang, Tan, Zhang, & Yao, 2015 ). To test the safety of PD, we administered a single large PD dose of 5000 mg/kg body weight to mice and observed no adverse effects. The LD50 value of PD is presumed to be greater than 5000 mg/kg body weight in mice, and PD can be effectively considered a non‐toxic substance.

PD has been shown to possess antioxidant and anti‐inflammatory activities (Mohan et al., 2013 ). Previous studies have demonstrated the therapeutic effects of PD on HG‐induced injury in multiple organs, including the heart, liver, and brain (Xu et al., 2016 ; Zhang et al., 2015 ). In the present study, we observed that PD decreased all of the analyzed markers of renal dysfunction (Table 2 ) in KKAy mice, suggesting that PD possesses renoprotective effects.

Recent studies have demonstrated that podocyte loss occurs in patients with DN (Maezawa, Takemoto, & Yokote, 2015 ). We observed that the shape of the foot processes, shape of the filtration slits, and the pore density of podocytes in KKAy mice can be restored after PD treatment (Figure 1 a–c). Nephrin and podocin are podocyte‐specific proteins that play crucial roles in the function of the glomerular filtration barrier. Nephrin is a key factor in the glomerular slit diaphragm. The absence of nephrin leads to proteinuria and foot process effacement (Teng et al., 2016 ). The expression of renal nephrin and podocin was significantly reduced in diabetic KKAy mice. Our data demonstrate that PD treatment restores the levels of nephrin and podocin proteins (Figure 1 d). These results strongly indicate that PD can reverse podocytes apoptosis in diabetic KKAy mice glomeruli.

To explore the protective effect of PD on podocytes further, we took advantage of MPC5 cells exposed to HG conditions to assess the effect of PD on cell apoptosis. HG has also been shown to induce podocyte apoptosis, and this effect has been attributed to the dephosphorylation of cytosolic phospho‐Bad and the associated accumulation of cytosolic cytochrome C (Yuan et al., 2017 ). In the present study, PD protected against HG‐induced MPC5 cell injury by inhibiting both cell apoptosis and the loss of slit diaphragm proteins. Furthermore, podocyte apoptosis induced by Aldo was accompanied by the loss of two silt diaphragm proteins, nephrin, and podocin. Our findings, demonstrate that PD protects against HG‐induced podocyte injury in vitro by inhibiting both cell apoptosis and the loss of slit diaphragm proteins (Figure 2 ).

During the progression of type 2 diabetes, mitochondria play a major role in podocyte apoptosis and the development of diabetes (Wang et al., 2012 ; Xu et al., 2016 ). Mitochondrial fission is a highly regulated process, and mitochondrial fragmentation is involved in the leakage of mitochondrial membrane proteins and the early stages of cell apoptosis (Zou, Roth, Younis, Burgoon, & Ganey, 2010 ). It is now widely recognized that mitochondrial dynamics are crucial for mitochondrial function, maintenance, and quality control (Westermann, 2010 ). Altered mitochondrial dynamics are often accompanied by increased ROS production, decreased cellular ATP and defects in cellular respiration evoked by oxidative damage (Ma, 2013 ). Mitochondrial dysfunction in podocytesis is increasingly recognized as a factor contributing to the pathogenesis of DN (Haas et al., 2016 ). To evaluate mitochondrial function, we selected a group of indicators, such as mitochondrial morphology, MMP, and ATP production. PD strongly prevented mitochondria fragmentation in HG‐induced podocytes, which suggests the therapeutic effect of PD on DN (Figure 3 ).

Drp1, a large GTPase, plays a critical role in mitochondrial fission. In agreement with previous findings (Liu et al., 2015 ), the level of Drp1 was markedly increased in hyperglycemic MPC5 cells, but this increase was significantly attenuated by PD treatment (Figure 4 a). We observed a similar effect of PD on Drp1 in KKAy mice; the level of Drp1 in mouse kidney was significantly decreased by PD treatment (Figure 4 b). Given that the phosphorylation status of Drp1 determines mitochondrial fission in podocytes, we also examined phosphorylated Drp1 (p‐Drp1) at serine residue 616 in MPC5 cells and primary podocytes. PD markedly attenuated the HG‐induced elevation of p‐Drp1 levels (Figure 4 ).

Moreover, the number of fragmented mitochondria in hyperglycemic MPC5 cells was significantly decreased with PD treatment. According to the literature, Drp1 is essential for the release of cytochrome C from mitochondria and for the activation of caspase‐3 (Hamacher‐Brady & Brady, 2015 ). However, it remains unknown whether Drp1 can induce podocyte apoptosis. Therefore, we infected MPC5 cells with either Drp1‐GFP lentivirus or Drp1 siRNA lentivirus to upregulate or downregulate Drp1 expression, respectively. Cell apoptosis and mitochondrial fission were increased by Drp1 upregulation; furthermore, Drp1 downregulation was associated with the inhibition of both mitochondrial fission and cell apoptosis (Figure 5 a,b). Goyal, Fell, Sarin, Youle, and Sriram, (2007 ) suggested that knockdown of Drp1 by siRNA delays but does not prevent HG‐induced cell apoptosis. In the present study, we observed apoptosis in podocytes transfected with Drp1‐GFP under hyperglycemic conditions (Figure 5 c–e), and this apoptosis was further blocked by PD treatment. Our result suggests that PD protects against HG‐induced podocytes apoptosis through a Drp1‐dependent mechanism.

We also demonstrated that PD prevents primary podocytes apoptosis secondary to antioxidant activity (Figure 6 ). A growing body of evidence suggests that excess cellular ROS plays a key role in mitochondrial perturbations, the pathogenesis of diabetes complications, and podocyte apoptosis (Khamaneh, Alipour, Sheikhzadeh Hesari, & Ghadiri Soufi, 2015 ). In the current study, we demonstrated that HG‐induced ROS can be significantly blocked by PD treatment. Furthermore, the expression of Drp1 was positively associated with the cellular ROS level (Figure 7 ), suggesting that PD may protect mitochondria through inhibition of ROS generation. Collectively, our findings provide new insights into the pathogenic process of HG‐induced podocyte injury and also identify a new therapeutic target of ROS/Drp1/mitochondrial fission/apoptosis pathway for DN.
